# Repeatability of signalling traits in the avian dawn chorus

**DOI:** 10.1186/s12983-019-0328-7

**Published:** 2019-07-09

**Authors:** Marc Naguib, Joris Diehl, Kees van Oers, Lysanne Snijders

**Affiliations:** 1 Behavioural Ecology Group, Wageningen University & Research, Wageningen, De Elst 1, 6708WD, Wageningen, The Netherlands; 2 Netherlands Institute of Ecology (NIOO-KNAW), Wageningen, The Netherlands; 3 Department of Evolutionary Ecology, Leibniz-Institute for Zoo and Wildlife Research, Berlin, Germany

**Keywords:** Animal communication, Bird song, Dawn chorus, Dawn song, Great tit, Behavioural repeatability, Singing activity, Song repertoire

## Abstract

**Background:**

Birdsong, a key model in animal communication studies, has been the focus of intensive research. Song traits are commonly considered to reflect differences in individual or territory quality. Yet, few studies have quantified the variability of song traits between versus within individuals (i.e. repeatability), and thus whether certain song traits indeed provide reliable individual-specific information. Here, we studied the dawn chorus of male great tits (*Parus major*) to determine if key song traits are repeatable over multiple days and during different breeding stages. Additionally, we examined whether repeatability was associated with exploration behaviour, a relevant personality trait. Finally, we tested if variation in song traits could be explained by breeding stage, lowest night temperature, and exploration behaviour.

**Results:**

We show that the start time of an individual’s dawn song was indeed repeatable within and across breeding stages, and was more repeatable before, than during, their mate’s egg laying stage. Males started singing later when the preceding night was colder. Daily repertoire size was repeatable, though to a lesser extent than song start time, and no differences were observed between breeding stages. We did not find evidence for an association between exploration behaviour and variation in dawn song traits. Repertoire composition, and specifically the start song type, varied across days, but tended to differ less than expected by chance.

**Conclusions:**

Our findings that individuals consistently differ in key song traits provides a better understanding of the information receivers can obtain when sampling songs of different males. Surprisingly, start time, despite being influenced by a highly variable environmental factor, appeared to be a more reliable signal of individual differences than repertoire size. Against expectation, singers were more repeatable before than during their mate’s egg laying stage, possibly because before egg laying, females are less constrained to move around unguarded and thus may then already sample (and compare) different singers. Combining repeated dawn song recordings with spatial tracking could reveal if the sampling strategies of receivers are indeed important drivers of repeatability of song traits. Such a complementary approach will further advance our insights into the dynamics and evolution of animal signalling systems.

## Background

Birdsong is a key model system in animal communication, playing an important role in mate attraction, territory defence, and the establishment and maintenance of social relations [1–3]. In the temperate zones, singing activity peaks at dawn in most species [4] and there are several mutually non-exclusive hypotheses on why birds sing this early in the morning [5, 6]. From a receiver perspective, the dawn chorus is particularly interesting as it is a reliable time to gather information from individual singers. Moreover, the dawn chorus provides immediate information on the spatial locations of conspecifics, allowing for direct comparison of singers in a communication and social network [7, 8], and to monitor territory occupancy [9].

The structural and temporal complexity of birdsong potentially provides a wealth of information to receivers. The timing of singing, for instance, might be indicative of the current condition of a singer, and it has been suggested that an early start time of singing may provide information about a bird’s current body condition, or more generally reflect individual or territory quality [5, 10, 11]. In contrast, production performance related traits (like trill rates and bandwidths) have been shown to reflect more fundamental neuromotor coordination abilities or age [12–15]. Learned components, such as repertoire composition and size, can also reflect age [16], yet have most frequently been shown to reflect conditions experienced during song learning [17–22] and might even be an indicator of general cognitive performance [23].

Many behavioural traits are known to consistently differ between individuals, i.e. they are ‘repeatable’, meaning that the expressed trait variation is smaller within individuals than between individuals [24]. Song traits that are repeatable therefore have the potential to provide the social environment with relevant information on the individual and territory quality of a singer. Yet, errors are inevitable in communication [25, 26] and singing behaviour does not only vary with stable individual and environmental characteristics, but also with varying environmental conditions and social interactions [7, 27–31]. Knowing what information is coded in song traits, and thus can be extracted by receivers, will help us understand how selection pressures, acting through the behaviour of these receivers, may shape animal communication systems.

Although studies on repeatability of dawn song traits have been limited so far [32], they have revealed that certain traits are repeatable across different sampling days. In eastern kingbirds (*Tyrannus tyrannus*), start time and song rate were repeatable between two successive years, yet start time was not repeatable within a given year and song rate was repeatable only within one of the years [10]. In great tits (*Parus major*), the start time and song rate were repeatable in the short-term, i.e. between two sample days within the egg laying stage [30]. A song trait like start time, however, may still be expected to harbour substantial within-individual variation, due to the known influences of environmental factors [33]. Therefore, song start time may be less repeatable than a more structural song trait like song repertoire [34].

Repeatability of repertoire size is especially interesting, because repertoire size is often considered to be a fitness-relevant signalling trait, i.e. a predictor of survival and/or reproductive output [15, 35–38]. The song repertoire of many species is learned early in life but may be modified later [39] and birds can still vary in decisions with respect to the songs they select from the repertoire. For instance, thrush nightingales (*Luscinia luscinia*) have been shown to adjust part of their repertoire to that of their neighbours [40]. Whether certain species can, and indeed will, modify their repertoire later in life is not always clear. Great tits, for example, were reported to frequently change their repertoire size and composition across years [41]. However, another study in great tits found these traits to be highly repeatable and similar [42]. Moreover, some species use different song types when they sing in response to other singing males in comparison to when they sing ‘undisturbed’ [43]. This was illustrated by nightingales (*Luscinia megarhynchos*), which were shown to sing specific song types only when they were exposed to them [44, 45]. Indeed, great tits exhibited a significant turnover in repertoire composition after exposure to unfamiliar song types [46], which could explain reduced repeatability. Although, another study [42] found great tits to be highly similar in their song repertoire compositions before and after exposure to unfamiliar song types. Thus, there remain substantial differences concerning the components and sources of variation in repertoire size and composition, both across and within bird species.

The repeatability of certain song traits might also, in itself, systematically vary among individuals [24, 47] or environments [48]. Such differences may be linked to differences in the motivation to sing consistently and may depend on breeding stage [49–51]. In addition, this could also relate to intrinsic differences between individuals, reflected by, for instance, personality traits. Certain personality traits, such as boldness and exploration behaviour, have been linked to the likelihood of an individual to alter its behaviour in response to changing circumstances. For instance, bold and fast exploring individuals are thought to be driven more by internal routines, and generally to be less responsive to external stimuli [52–54]. Consequently, these individuals can be expected to be more repeatable (i.e. less flexible); varying their song less over time and across conditions. Additionally, personality might not only be related to within-individual variation in singing behaviour but also to variation in behaviour between individuals. Personality traits have been shown to explain variation a range of different behaviours including territorial, spatial and social behaviour in songbirds [55–60] and to be associated with ‘undisturbed’ singing in some cases [50, 61, 62] but not in others [30, 63]. Detailed information of between- and within-individual variation of specific song traits, including the potential drivers of this variation, will lead to a better understanding of the information received by conspecifics. This is especially relevant when competitor assessments and mate choice decisions involve sampling of different singers on a variety of days [9, 64, 65].

Here, we determined the repeatability of specific dawn song traits using the most studied avian model system in the wild, the great tit [66]. By using automated dawn song recordings in a great tit nest box population, we collected song data from 25 males over several days and during two distinct breeding stages (three to twelve recordings per male). We focused on dawn song start time, representing an instantly available signal trait to receivers, and which was previously shown to be variable, yet significantly repeatable within the egg laying stage [30]. Repertoire size is a more complex and learned trait, which we expected to be less variable over time. We considered overnight temperature as a potential short-term driver of variation, with cold nights negatively affecting a singer’s energy level at dawn and thereby its singing behaviour [33, 67–69]. We considered the breeding stage as a potential longer-term driver of within-individual variation, with the onset of egg laying providing a distinct switch to a more standardized breeding context. We expected that singing of males would be more repeatable during egg laying than before egg laying. Egg laying provides a more standardized context with the onset of breeding being distinctively evident and females are fertile during egg laying leading to important reproductive decisions during this time. Finally, for a subset of the males, we examined whether there was evidence for faster-exploring and thus more routine-prone individuals, to be more repeatable in song traits (i.e. show less within-individual variation in their singing behaviour). Additionally, we tested for a correlation between a male’s exploration score and the absolute song trait values we analysed (i.e. start time and song repertoire).

## Results

### Start time

**Fig. 1:**
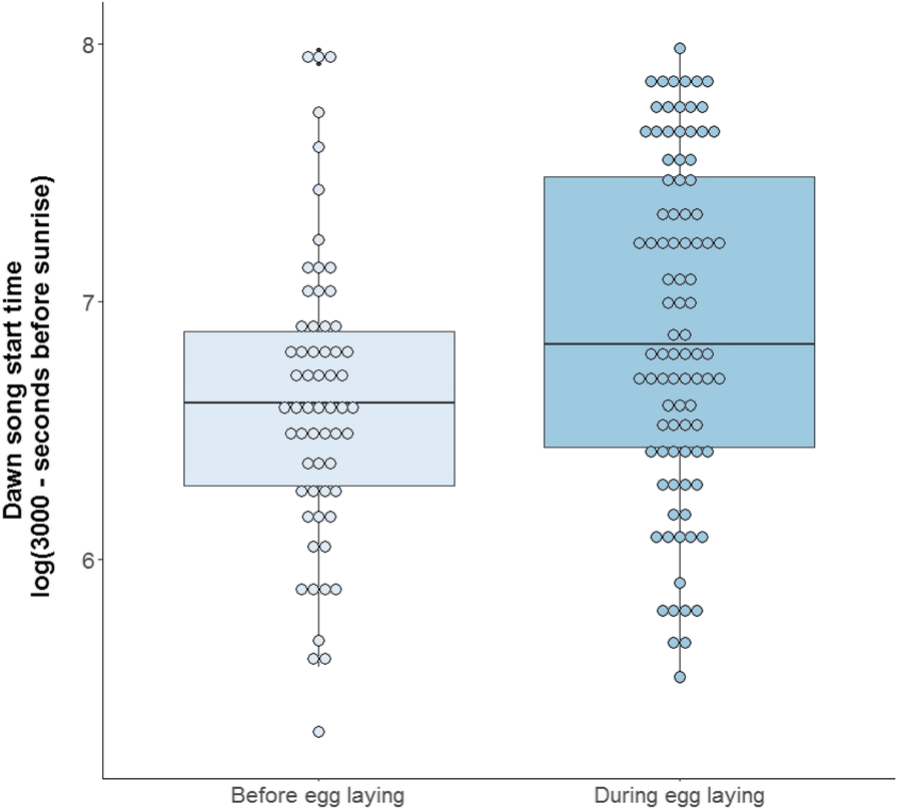
Dawn song start time in relation to breeding stage. Individual dots visualize raw data values (i.e. one value for a recording per individual per day). The maximum dawn song start time was 2793 s and the minimum 77 s before sunrise (*Mean* +/− *SD*: 1923 +/− 684). Start time in seconds before sunrise was inverted (using a round number close to the maximum start time) and log-transformed

**Fig. 2:**
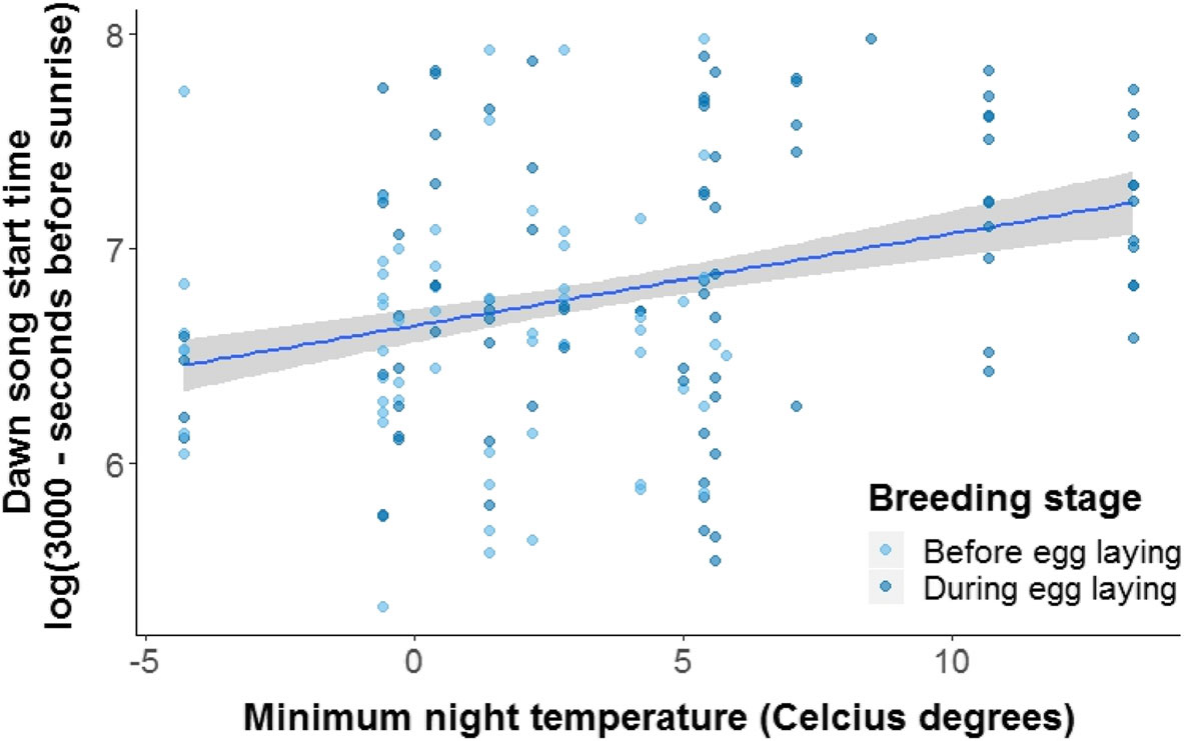
Dawn song start time in relation to minimum night temperature. Lighter dots show the start times before the egg laying stage and darker dots during the egg laying stage. Regression lines and *95% CI* (shaded area) are based on fitted model values. The maximum dawn song start time was 2793 s and the minimum 77 s before sunrise (*Mean* +/− *SD*: 1923 +/− 684). Start time in seconds before sunrise was inverted and log-transformed

The time that individual males started their dawn song was repeatable over multiple days (*R* = 0.41, Table 1). Males were significantly more repeatable in their start time before egg laying than during egg laying (Table 1, non- overlapping *84% CI*). Moreover, the repeatability in start time of the dawn song by slow explorers did not differ from the repeatability in start time by fast explorers (Table 1, overlapping *84% CI*). The actual time a male started his dawn song did not differ significantly between breeding stages (before or during egg laying) (LMM, *Estimate* ± *SE* = 0.05 ± 0.10, *χ*^*2*^ = 0.26, *P* = 0.61, *N*_*ind*_ = 25*, N* = 152, Fig. 1) and was not correlated with exploration score (LMM, *Estimate* ± *SE* = 0.007 ± 0.01, *χ*^*2*^ = 0.30, *P* = 0.58, *N*_*ind*_ = 18, *N* = 111). Males started singing significantly later when the minimum nightly temperature (which strongly correlated to current temperature; see Methods) was lower (LMM, *Estimate* ± *SE* = 0.04 ± 0.01, *χ*^*2*^ = 8.43, *P* = 0.004, *N*_*ind*_ = 25*, N* = 152, Fig. 2).

**Table 1:** Individual repeatability estimates for start time of dawn song (seconds before sunrise) and dawn song repertoire size

Dawn song trait		R	95% CI	84% CI	V_ind_	V_resid_	N_ind*_	N_rec**_	P_perm_
Start time	all birds	0.41	0.22–0.57	0.27–0.53	0.17	0.18	25	152	**0.001**
	before egg laying	0.67	0.38–0.83	**0.48–0.79**	0.24	0.11	18	57	**0.001**
	during egg laying	0.31	0.10–0.53	**0.14–0.47**	0.14	0.22	22	91	**0.001**
	slow explorer	0.36	0.06–0.61	0.13–0.54	0.11	0.15	10	65	**0.001**
	fast explorer	0.59	0.18–0.81	0.29–0.75	0.25	0.14	8	46	**0.001**
Repertoire size	all birds	0.21	0.004–0.33	0.05–0.30	0.08	0.31	25	150	**0.001**
	before egg laying	0.12	0.00–0.32	0.00–0.24	0.04	0.27	17	55	0.17
	during egg laying	0.20	0.00–0.37	0.00–0.30	0.08	0.33	22	90	**0.03**
	slow explorer	0.06	0.00–0.22	0.00–0.14	0.02	0.31	10	63	0.15
	fast explorer	0.03	0.00–0.19	0.00–0.12	0.01	0.23	8	46	0.23

*Number of individuals, only including individuals with at least two recordings for the given condition **Total number of recordings

Among and within group variance estimates are reported as *V*_*ind*_ and *V*_*resid*_ respectively. *P*-values are based on 1000 permutations. Non-overlapping 84% confidence intervals indicate significant differences between group repeatability estimates. Significant values are highlighted in bold

### Repertoire size

**Fig. 3:**
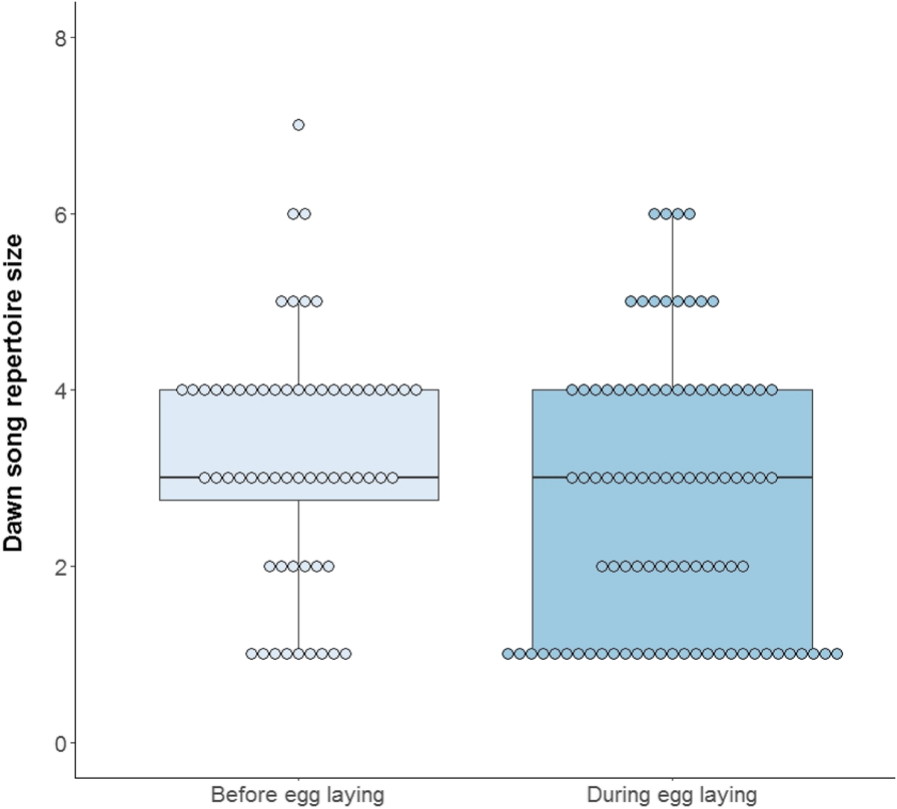
Dawn song repertoire size in relation to breeding stage. Individual dots show raw data values (i.e. one value for a recording per individual per day)

The full repertoire size, calculated from all recordings of a given male, including song types following the dawn song (i.e. also after a ≥ seven-minute break in singing, see Methods), ranged from three to seven song types per male (*Mean* ± *SD* = 4.72 ± 1.17). The full dawn repertoire size calculated from all recordings of a given male, but which excluded song types following the dawn song, ranged very similarly from two to seven song types per male (*Mean* ± *SD* = 4.32 ± 18). The number of distinctive song types sang at dawn by a male on any given morning (dawn song repertoire) ranged from one to seven and was significantly repeatable (*R* = 0.21, Table 1). Repeatability of the dawn song repertoire before egg laying did not differ from that during egg laying (Table 1, overlapping *84% CI*). Also, slow explorers did not differ from fast explorers in the repeatability of their dawn song repertoire (Table 1, overlapping *84% CI*). The number of song types a male sang during a given dawn song did not differ significantly between breeding stages (GLMM, *Estimate* ± *SE* = − 0.12 ± 0.11, *χ*^*2*^ = 1.06, *P* = 0.30, *N*_*ind*_ = 25, *N* = 150, Fig. 3) and was not correlated to exploration score (GLMM, *Estimate* ± *SE* = 0.01 ± 0.01, *χ*^*2*^ = 1.53, *P* = 0.22, *N*_*ind*_ = 18, *N* = 109). Minimum nightly temperature had no significant association with dawn song repertoire size (GLMM, *Estimate* ± *SE* = − 0.01 ± 0.01, *χ*^*2*^ = 0.198, *P* = 0.32, *N*_*ind*_ = 25, *N* = 150).

### Repertoire composition

**Fig. 4:**
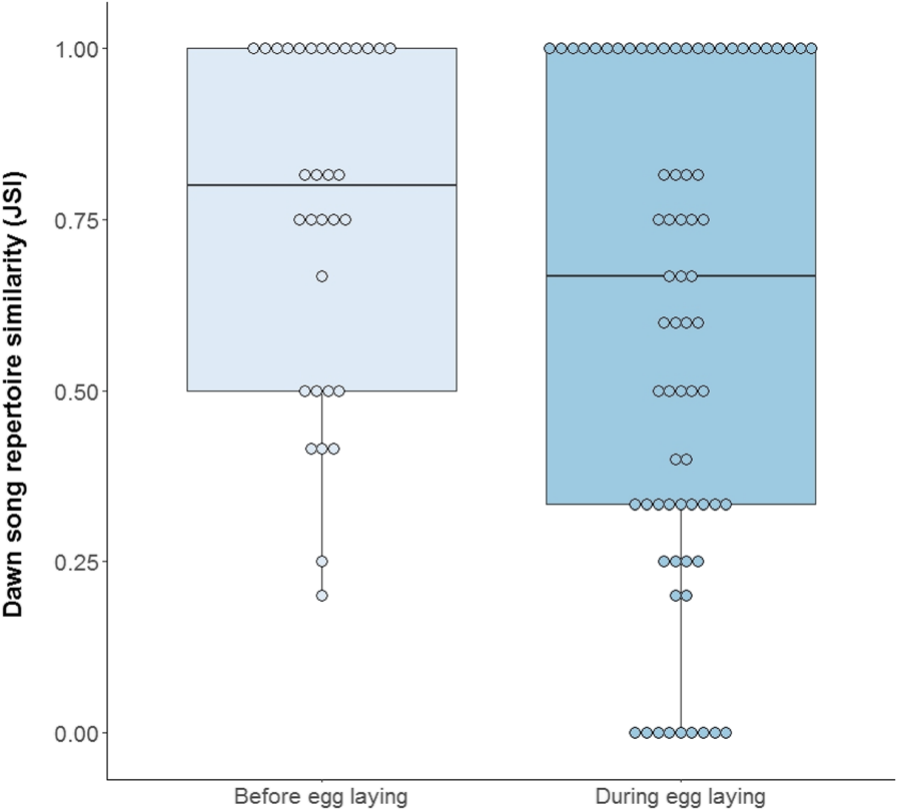
Dawn song repertoire similarity in relation to breeding stage. Individual dots visualize raw data values of recordings made on consecutive days (compared within individual). Similarity in dawn song repertoire was calculated using the Jaccard’s Similarity Index (JSI)

The dawn song repertoire composition of individual singers was relatively similar between consecutive days. The median similarity coefficient (SC), based on consecutive days within breeding stages, was 0.75 (*Min* = 0.00, *Max* = 1.00, *84% CI*: 0.60–0.80, *N*_*ind*_ = 24, *N* = 103). Dawn song repertoire similarity was similar before egg laying (SC = 0.80, *84% CI*: 0.67–1.00, *N*_*ind*_ = 15, *N* = 32) and during egg laying (SC = 0.67, *84% CI*: 0.50–0.80, *N*_*ind*_ = 20, *N* = 71), and thus did not significantly differ between these breeding stages (GLMM, Estimate ± SE = − 0.48 ± 0.55, *χ*^*2*^ = 0.79, *P* = 0.38, *N*_*ind*_ = 24, *N* = 103, Fig. 4). Dawn song repertoire similarity was also similar for slow (SC = 0.75, *84% CI*: 0.60–1.00, *N*_*ind*_ = 10, *N* = 43) and fast explorers (SC = 0.80, *84% CI*: 0.50–1.00, *N*_*ind*_ = 8, *N* = 34). Indeed, there was no correlation between dawn song repertoire composition similarity and exploration score (GLMM, *Estimate* ± *SE* = − 0.02 ± 0.05, *χ*^*2*^ = 0.16, *P* = 0.69, *N*_*ind*_ = 18, *N* = 77). Minimum nightly temperature was not correlated with dawn song repertoire similarity (GLMM, *Estimate* ± *SE* = − 0.02 ± 0.05, *χ*^*2*^ = 0.12, *P* = 0.73, *N*_*ind*_ = 24, *N* = 103).

### Start song type preference

The song type used to start the dawn song varied across consecutive days and changed 68% of the time over 105 dawn song comparisons. However, there tended to be fewer changes in start song type than expected (*V* = 86, *P* = 0.07, *N*_*ind*_ = 24), based on the full repertoire available to each male. The number of changes of the start song type did not differ between breeding stages (GLMM, *Estimate* ± *SE* = − 0.31 ± 0.59, *χ*^*2*^ = 0.27, *P* = 0.60, *N*_*ind*_ = 24, *N* = 105) and slower explorers were not more likely to change their start song type than faster explorers (GLMM, *Estimate* ± *SE* = 0.03 ± 0.03, *χ*^*2*^ = 0.75, *P* = 0.39, *N*_*ind*_ = 18, *N* = 79). Minimum nightly temperature was not correlated with individual propensity to change start song type (GLMM, *Estimate* ± *SE* = − 0.03 ± 0.05, *χ*^*2*^ = 0.38, *P* = 0.54, *N*_*ind*_ = 24, *N* = 105).

## Discussion

Here we show that key song traits during the dawn chorus were repeatable, i.e. varied more between than within individuals, over several days and across breeding stages. Males also tended to vary their start song type less than expected by chance. These key traits could thus be used by receivers as a source of information of stable individual characteristics or stable environmental conditions.

Interestingly, dawn song start time, despite also being influenced by a variable environmental factor like overnight temperature, appeared to be more repeatable and thus more suitable for receivers to predict individual or territorial characteristics, than a learned trait such as repertoire size. Relatively low (yet significant) repeatability estimates generally indicate that receivers would need to obtain multiple samples per individual to obtain a reliable estimate of how a specific singer compares to other singers in the social environment. This is especially relevant for receivers that are restricted in the number of samples they can take, such as prospecting birds, which might stay only briefly in a given area [9, 64, 70]. Understanding which signalling traits are readily accessible to receivers is essential to elucidate how potential receivers (i.e. social mates, extra-pair mates and/or competitors) might sample their social environment and, vice versa, how the sampling strategies of receivers might shape the production and repeatability of signals.

Previous studies have shown that the time a bird starts to sing during the dawn chorus is related to social factors [29, 71], environmental factors, such as artificial and natural lighting [72, 73], and noise levels [74]. In addition, overnight temperature can be more variable than light levels or noise and was previously shown to be associated with the duration of dawn song in great tits [67]. Here, we show that overnight temperature was significantly associated with the start time of dawn song in male great tits. Nonetheless, individuals were significantly repeatable in their start time, independent of whether or not we controlled for minimum night temperature in the model (see Methods). In other words, some individuals consistently started singing earlier than others, even when overnight temperatures were low. Notably, minimum nightly temperature strongly correlated with temperature at sunrise, leaving open the possibility that current temperature, rather than overnight temperature, was the proximate environmental factor influencing singing behaviour. Yet, both overnight and current ambient temperature are expected to affect energy expenditure. Singing earlier might thus be a reliable signal of a singer’s body condition and thereby possibly also a singer’s territory quality [68, 69]. In support of this hypothesis, a study on eastern kingbirds showed that earlier singers indeed gained a reproductive benefit, by being paired to earlier breeding females, which also (in one year) laid larger clutches [10] and a study on Lincoln’s sparrows (*Melospize lincolnii*) showed females to prefer songs sung in the cold [75]. Additionally, a study on blue tits (*Cyanistes caeruleus*) revealed that earlier singers, who were also older, had more sexual partners and were more likely to gain extra-pair paternity [76]. Yet, the relevance of start time as a song trait reflecting potentially fitness-relevant information has received surprisingly little attention compared to other song traits.

Repeatability in dawn song start time, surprisingly, was higher before egg laying than during egg laying. We predicted males to be more repeatable during the egg laying stage, because we presumed that during this time, when the female is fertile and the onset of breeding is distinctively evident for the male, important fitness-dependent decisions are made and that it thus would be important for males to sing more consistently. Indeed, dawn singing activity in great tits peaks during the egg laying stage [50, 77], even though high song output is not restricted to this period [50, 78]. Yet, female great tits may make their reproductive decisions well before they become fertile. This is obviously evident when it comes to choosing a social partner, but might also apply for whether, and with whom, to engage in extra-pair activities. If females indeed need to sample multiple males several times to obtain a good estimate of their individual quality relative to other males, it would be likely that this would begin already before egg laying. This would make the before egg laying stage a highly relevant time for her social partner (our singing subject) to distinguish himself from other singers and might be a factor explaining the high singing activity also at this stage [50]. In several other bird species, the time before egg laying indeed appeared to be the most relevant singing time, with male pied flycatchers (*Ficedula hypoleuca*) and chaffinches (*Fringilla coelebs*) significantly decreasing in several aspects of their singing performance immediately after pairing [49, 51]. Alternatively or additionally, the process of egg laying itself, which occurs during dawn, may restrict a female in the time and energy she can spend on sampling potential sexual partners, generating less selective pressure for singers to maintain a high-level singing performance during this period.

We presumed that, given the small repertoire size of great tits (generally clearly below 10 song types), males would display their full repertoire each morning, resulting in high similarity and repeatability estimates. Dawn repertoire size was indeed significantly repeatable, yet the repeatability estimate was lower than we had expected (i.e. *R* = 0.21). Repertoire similarity was relatively high between successive dawns (i.e. median SC = 0.75), but regularly ranged from completely identical to completely different. Similarly, although males tended to start with the same song type more often than expected, we recorded a change in starting song type 68% of the time. Such changes in repertoire and starting type across days may function in maintaining receiver interest by displaying unexpected singing variation [79]. Alternatively, great tits may adjust their repertoire to the social context [46]. For example, singers may fine-tune their dawn song in response to what competitors are singing [43].

Our findings, in part, contrast a previous study showing that repertoire size did not change over years [42]. Differences between studies could be partly related to differences in methodology (e.g. automatic versus manual recording), but also to differences among populations [42]. It would be interesting to apply different recording methods to the same population to investigate this. Additionally, it would be intriguing to conduct a cross-population study on the repeatability of certain song traits, using the same methodology, and explore which population-level factors (e.g. population density) might explain between-population differences in repeatability estimates. Males singing in less populated areas may be more consistent in their displayed repertoire compositions than males singing in neighbourhood with higher population densities and thus, presumably, higher social responsiveness [30].

We expected that fast exploring individuals, who are commonly assumed to follow more routines [80], would be more repeatable in their dawn song traits compared to slower individuals, yet we did not find evidence for this. We also did not find evidence for a direct relationship between exploration behaviour and song trait values. It should be stressed, however, that we only had a modest number of personality-tested individuals in our study and only one exploration score per individual. As a consequence, we lack the statistical power to draw solid conclusions from these findings and this aspect of our study should therefore be viewed as exploratory. Yet, given the difficulty of obtaining personality data and repeated song recordings for free-ranging individuals, we consider our findings still valuable and relevant in light of future review studies and meta-analyses on this topic [81, 82]. In addition, our previous study in the same population, also did not find a relationship between exploration behaviour and variation in dawn song traits [63]. Studies examining behaviour in response to a territory intrusion (i.e. a confrontational context), in contrast, revealed relationships between exploration behaviour, several song traits and movement patterns [55, 56, 63]. Together, these results suggest that personality-related differences in singing behaviour may be revealed in only specific song traits and/or in response to stressful challenges, rather than in the relatively undisturbed context of the dawn chorus.

## Conclusions

We showed repeatability and similarity of commonly studied song traits across fourteen days and two breeding stages in a wild songbird population. Despite variable environmental conditions, individuals consistently differed from each other in their dawn song performance. Yet, at least one of the song traits also flexibly varied with breeding stage and ambient temperature. Future studies investigating receiver behaviour in relation to variation in these song traits are likely to shed additional light on the information value of these traits and thereby the potential selection pressures acting on them.

## Methods

### General

The study was conducted between 26 April and 15 May 2013 at Westerheide, a mixed forest near Arnhem, The Netherlands (52.016000, 5.841000). The area covers circa 120 ha with 200 nest boxes with approximately 75 to 100 great tit breeding pairs per year. Great tits here are caught at regular intervals the whole year round, through a routine procedure with mist nets at feeders or in the evening during roosting checks. When individuals are caught for the first time they are provided with a uniquely numbered aluminium ring and taken indoors to test their exploration behaviour, an established operational measure of an avian personality trait [83]. The novel environment tests are performed throughout the year until the start of the breeding season. After the birds are caught, they are brought to the laboratory where they are kept overnight in individual cages (0.9 × 0.4 × 0.5 m), provided with mealworms and ad libitum water, sunflower seeds, and a commercial seed mixture. The next morning, the birds are tested for their exploration behaviour in a closed room (4.0 × 2.4 × 2.3 m) with five artificial trees [84]. Birds enter the test room through a sliding door in their cage. The total number of flights between trees and the hops within trees within two minutes is used to calculate an overall exploration score [85]. Faster explorers receive a higher score than slower explorers. In the afternoon after the test, the birds are released near their site of capture after testing. We did not have repeated scores of exploration behaviour for sufficient subjects to quantify the within-individual variation in exploration behaviour. However, our measure of exploration behaviour was previously shown to be repeatable in this study population [85, 86]).

### Song recordings and song analysis

We used time-programmable song recorders (SM2 song meter, Wildlife Acoustics Inc. Maynard, MA, U.S.A. and time-programmable Olympus DM650 and DM670 audio recorders) to record dawn song at the breeding nest box on up to 12 days per male. During the nest building and egg laying breeding stages, great tit males commonly sing near the nest box in which their mate is roosting at dawn. We therefore placed a song recorder 1.5 m above nest boxes with nest building activity (i.e. accumulation of pieces of moss in the nest box). We programmed each recorder to start recording from approximately one hour before sunrise (range: 53–66 min before sunrise) and to continue recording for four hours. Each subject was assigned its own recorder and only one singing individual per recorder was analysed (i.e. more distant individuals singing were not considered). Songs were recorded as *wav* files with a sampling rate of 44.1KHz and 16 bit sampling accuracy. We recorded 25 males for several days between seven days before onset of egg laying and seven days from the onset of egg laying (“before” and “during” egg laying hereafter). In total we made 179 recordings (three to 12 dawn recordings per male), of which three recordings contained no song, 24 recordings contained a bird starting only after sunrise (Mean = 2996 s, range 255 s to 7935 s after sunrise) and six recorders stopped prematurely (for two of these recordings only the start time and start song type could be reliably determined). Two recordings were made after the last egg was laid. We could determine the identity of 20 males by catching them during the nestling provisioning stage [87]; 18 of these males were previously personality tested. Daily sunrise data and minimum sunrise and nightly temperature (between 00:00 and 06:00) were retrieved from the KNMI (Koninklijk Nederlands Meteorologisch Instituut). Minimum nightly temperature was highly correlated to temperature at sunrise and thus also reflected the relative temperature during the time a male was singing (*r* = 0.74, *95% CI* = 0.43–0.89, *P* < 0.001).

The song recordings were analysed using Avisoft-SASLab Pro, Version 5.1 (Avisoft Bioacoustics, Berlin, Germany). In order to obtain a performance-derived definition for the end of an individual’s dawn song in our recordings [88], we first measured all inter-song pauses before sunrise for 16 individuals. Of all inter-song pauses measured, 98% were shorter than seven minutes. Based on this inter-song interval distribution, for the subsequent analyses, we defined the dawn song of an individual until a male stopped singing for longer than seven minutes, even if the singing ended after dawn (after sunrise). This definition, derived from the actual singing behaviour near the nest box, is likely to reflect a similar endpoint as those using female emergence from the nest box, as males then usually interrupt or stop singing [89]. Two third of all the dawn songs ended before sunrise. The birds that continued singing also after sunrise (i.e. without a seven-minute pause), did so for a maximum of 22 min after sunrise, but for eight minutes on average. For every dawn song recording the (1) start time (seconds before sunrise), (2) start song type, (3) song repertoire size and (4) song repertoire composition was determined. The dawn repertoire size was defined as the number of distinctive song types sang by a subject during its dawn song on a given day (quantitative measure of repertoire) and dawn repertoire composition was defined as the combination of song types used at dawn on a given day (qualitative measure of repertoire). The full dawn repertoire size was the number of distinctive song types sang, combining all dawn song recordings of a given male, but excluding song types sang after a seven-minute pause. The full song repertoire size was the number of distinctive song types sang, combining all song recordings of a given male, including song types sang after a seven-minute pause. Distinctive song types could clearly be distinguished based on sound spectrograms.

### Statistical analysis

All analyses were done in R 3.4.1. for Windows (R Core Team, 2017). We constructed linear (LMM) and generalized mixed models (GLMM) using the ‘lmer()’ and ‘glmer()’ functions of the *lme4* package [90]. Individual identity and recording date were included as random factor in all models. Fixed effects were tested by comparing the model including the variable of interest (e.g. breeding stage, minimum nightly temperature or exploration score) to the model excluding it (Likelihood ratio test). Because we did not know the exploration score of seven males, we left this variable out of the model when testing for fixed effects of breeding stage and temperature, allowing us to make full use of the sample size and maximize our test power. As a consequence, we thus conducted multiple tests (2 × 2). Applying a multiple testing correction would, however, not have changed our conclusions. Model fit was evaluated by visual inspection of the residual frequency distribution and the predicted values vs the residual plot or by testing for overdispersion.

#### Breeding stage, night temperature and exploration score

To test if there was an association between start time of an individual’s dawn song (continuous variable) or dawn repertoire size (count variable) and breeding stage (before or during egg laying), minimum nightly temperature (continuous variable) or exploration score (continuous variable), we constructed a LMM and a GLMM (Poisson distribution), respectively. We transformed start time to meet the linear model assumptions using a round number close to the maximum start time: *log (3000 - s before sunrise).*

We analyzed variance in repertoire composition by calculating similarity coefficients (SC) for successive days using the Jaccard’s Similarity Index: SC = Z/((X + Y)-Z), with X and Y being the number of song types of day x and y, and Z being the number of shared song types [91]. For our analysis we only calculated the SC for successive days within breeding stages. Because of a high occurrence of 0 and 1 SC values, we transformed this measure to a binary variable, based on the *Median* SC value of 0.75 (*Min* = 0.00, *Max* = 1.00, *N*_*ind*_ = 24, *N* = 103), i.e. lower or equal to 0.75 = 0 and higher than 0.75 = 1. Confidence Intervals (*CI*) of Medians were calculated using the ‘MedianCI()’ function of the package *DescTools* [92]. To test for an association between the similarity coefficient and breeding stage, minimum nightly temperature or exploration score, we constructed a GLMM (Binomial distribution), again with breeding stage, minimum nightly temperature and exploration score as fixed effects and individual identity and recording date as random effects.

Finally, to determine if a male had a start song type preference, we calculated the observed and the expected number of changes in the start song type between successive days “((full repertoire size - 1)/full repertoire size)*(recording days - 1)”. Subsequently, we tested if the observed number of changes in start song type differed from expected by using a Wilcoxon Signed Ranks test. To test for an association between the number of changes and breeding stage or exploration score, a GLMM (Binomial distribution) was constructed, including the same fixed and random effects as stated above. Because we did not calculate similarity indices and start song type changes between recordings that crossed breeding stages and/or were not consecutive (i.e. made on the next day), sample sizes for these analyses differ from start time and repertoire size analyses.

#### Repeatability of start time and dawn repertoire size

Individual repeatability of start time and song repertoire size was quantified using the functions ‘rptGaussian()’ and ‘rptPoisson()’ of the package *rptR* [93], with individual identity as random effect of interest, but also including recording date as random effect. Fixed effects were not included when calculating the reported repeatability (*R*) values. Including minimum nightly temperature in the model for start time slightly increased repeatability for this trait (*R*_*adj*_ = 0.45, *95% CI* = 0.25–0.61, *P*_*perm*_ = 0.001). We conducted 1000 bootstraps and 1000 permutations for each repeatability estimate. Reported *P*-values are based on permutation tests. Among (***V***_***ind***_) and within group (***V***_***resid***_) variance estimates were retrieved using the ‘re_var()’ function of the *Sjstats* R-package [94].

To test if repeatability changed with the onset of egg laying, the male song recordings (*N* = 25 individuals) were divided into two groups according to the breeding stage of the mate (before egg laying: *N* = 18 individuals, two – six dawn recordings/individual; during egg laying: *N* = 22 individuals, two – seven dawn recordings/individual), and repeatability was calculated separately for these groups. The same was done for exploration behaviour, where males were divided into two groups (high and low exploration score, *N* = 18 individuals). We used a cut-off value of an exploratory score of 17 based on the median value in our total dataset of > 10,000 tests. Individuals with scores lower than 17 are considered ‘slow explorers’ (*Median* = 15, *Min* = 6, *Max* = 16, *N* = 10 individuals), and individuals with 17 or higher are considered ‘fast explorers’ (*Median* = 23, *Min* = 19, *Max* = 39, *N* = 8 individuals). Given the small sample sizes for the comparison of personality types, this part of the analysis should be viewed as exploratory (i.e. no strong conclusions can be drawn on the basis of this single test). Following earlier studies [95, 96], repeatability levels (and song repertoire similarity coefficients scores) of groups were considered significantly different when 84% confidence intervals did not overlap each other. This criterium was based on the recommendation made by Julious (2004), who argued that absence of overlap between two 84% confidence intervals is equivalent to a 95% confidence interval around the difference not including zero [97]. Conclusions were the same for group comparisons using either 84% or 95% confidence intervals, with the exception of repeatability of start time which was considered significantly different between the before and during egg laying groups when using 84% but not when using 95% confidence intervals. Average repeatability of start time across breeding stages was *R* = 0.37 (*95% CI* = 0.18–0.58, *N*_*ind*_ = 19, *N* = 127), calculated by including breeding stage as a random slope term [98, 99] and only including individuals that were recorded during both breeding stages. The slope intercept correlation was 0.56, yet given that AIC was lower for the model with the random slope term (AIC = 216.42) compared to the model without (AIC = 220.30), this correlation should probably be regarded as uninformative.

## Data Availability

The dataset generated and analysed during the current study is available in the Dryad repository, [10.5061/dryad.r1s93g8].
